# Enhancing Photoelectrocatalytic Efficiency of BiVO_4_ Photoanodes by Crystal Orientation Control

**DOI:** 10.3390/nano14231870

**Published:** 2024-11-21

**Authors:** Hongru Zhao, Xinkong Wei, Yue Pei, Weihua Han

**Affiliations:** 1 Guangzhou Institute of Blue Energy, Guangzhou 510555, China; zhaohr21@lzu.edu.cn; 2 School of Physical Science and Technology, Lanzhou University, Lanzhou 730000, China; 220220939981@lzu.edu.cn (X.W.); pyue2023@lzu.edu.cn (Y.P.)

**Keywords:** photoelectrocatalysis, crystal facet engineering, carrier mobility, charge separation efficiency

## Abstract

Bismuth Vanadate (BiVO_4_) is a promising photoanode material due to its stability and suitable bandgap, making it effective for visible light absorption. However, its photoelectrocatalytic efficiency is often limited by the poor transport dynamics of photogenerated carriers. Recent research found that varying the atomic arrangement in crystals and Fermi levels across different crystal orientations can lead to significant differences in carrier mobility, charge recombination rates, and overall performance. In this work, we optimized the atomic arrangement by controlling the crystal growth direction to improve carrier separation efficiency using a wet chemical method. Systematic investigations revealed that the preferential [010]-oriented BiVO_4_ film exhibits the highest carrier mobility and photocurrent density. Under an applied bias of 1.21 V (vs. RHE) in a 0.5 M Na_2_SO_4_ electrolyte, it achieved a photocurrent density of 0.2 mA cm^−2^ under AM 1.5 G illumination, significantly higher than that of the [121]-oriented (0.056 mA cm^−2^) and randomly oriented films (0.11 mA cm^−2^). This study provides a deeper understanding of the role of crystal orientation in enhancing photoelectrocatalytic efficiency.

## 1. Introduction

Photoelectrocatalysis offers a promising solution to the global energy crisis and environmental challenges by converting solar energy into stable chemical energy stored in energy-dense molecules [1,2,3]. Metal oxides are ideal candidates for photoelectrocatalytic materials due to their high stability, low cost, and availability [4]. The photoelectrochemical (PEC) process involves three crucial steps: light absorption, separation and transport of photogenerated carriers, and surface redox reactions. Among these, the transport and separation of carriers often limit overall PEC efficiency [5,6,7]. In most metal oxides, the short diffusion length of photogenerated carriers leads to recombination before they can reach the surface, severely hindering PEC performance [8]. For instance, hematite has a light penetration depth of approximately 118 nm at 550 nm, which is significantly longer than its hole diffusion length of only 2–4 nm [9]. Similarly, BiVO_4_ is limited by a hole diffusion length of less than 40 nm, which constrains its photocurrent generation [10]. Even in widely studied materials, like polycrystalline TiO_2_, the solar-to-fuel conversion efficiency remains restricted due to short minority carrier diffusion lengths ranging from 10 to 100 nm [11].

Enhancing carrier diffusion length and separation efficiency is thus a critical challenge. While research has focused on increasing carrier mobility within photoelectrodes, grain boundaries and short hole diffusion lengths relative to light absorption create obstacles. Recent studies suggest that controlling crystal orientation can significantly improve carrier mobility and reduce recombination [12,13,14]. For example, Pan et al. demonstrated that Cu_2_O films grown along the [111] orientation exhibit a carrier mobility of 15.4 cm_2_ V^−1^ s^−1^, which is significantly higher compared to films with [100] (1.29 cm^2^ V^−1^ s^−1^) and [110] (0.87 cm^2^ V^−1^ s^−1^) orientations [15]. Similarly, Kay et al. found that the conductivity of hematite (α-Fe_2_O_3_) along the [110] orientation was four orders of magnitude higher than in the orthogonal direction [16]. These findings underscore the potential of crystal facet engineering to enhance carrier mobility and PEC activity [17,18,19,20].

BiVO_4_, with its narrow bandgap (2.4 eV), negative conduction band edge (0 V vs. RHE), earth abundance, non-toxicity, and excellent stability, has emerged as a prominent material for photoelectrocatalysis [21,22,23,24]. However, current methods to improve BiVO_4_ film quality require high vacuum, high temperatures, and specialized equipment. In this work, we introduce a novel strategy for improving BiVO_4_’s photoelectrocatalytic performance by controlling the crystal growth orientation of the material. This approach, which utilizes a simple wet chemical method, operates under ambient pressure, making it more cost-effective and scalable compared to conventional high-temperature or high-vacuum techniques. By tuning the growth temperature, we achieve BiVO_4_ films with distinct crystal orientations, significantly enhancing the material’s carrier mobility and photocurrent density.

In this study, we successfully prepared BiVO_4_ films with controlled crystal growth orientations using a wet chemical method by regulating the water bath temperature. This approach allows for film growth under ambient pressure while maintaining excellent stability during PEC processes. Our results show that BiVO_4_ photoanodes grown along the [010] crystal orientation achieve significantly higher carrier separation efficiency and photocurrent density compared to other orientations. Specifically, the photocurrent density of [010]-oriented BiVO_4_ is 3.5 times higher than that of the [121] orientation and twice that of films with no specific orientation. Conductive atomic force microscopy (C-AFM) testing and fitting confirm that [010]-oriented BiVO_4_ exhibits superior carrier mobility and diffusion length, offering an effective strategy for enhancing carrier separation efficiency in photoelectrodes.

## 2. Materials and Methods

### 2.1. Chemicals and Reagents

Bi(NO_3_)_3_·5H_2_O (AR, 99%) and NH_4_VO_3_ (AR, 99%) were acquired from Aladdin Chemical Reagent Co., Ltd. (Shanghai, China). HNO_3_ (AR), acetone (AR), and ethanol (AR) were purchased from Chengdu Kelong Chemical Reagent Co., Ltd. (Chengdu, China). Acetonitrile (AR) was obtained from Tianjin Damao Chemical Reagent Factory, and Fluorine-doped tin oxide (FTO) glass was purchased from Huanan Xiangcheng Technology Co., Ltd. (Shenzhen, China).

### 2.2. Preparation of the Samples

The synthesis of BiVO_4_ films follows a solution-based method combined with controlled annealing. First, 1.21 g of Bi(NO_3_)_3_·5H_2_O and 0.292 g of NH_4_VO_3_ were separately dissolved in 50 mL of HNO_3_ solution with a pH of 0.04, stirred for 30 min until fully dissolved. The two solutions were then combined, followed by the addition of 5 mL of acetonitrile, and stirred for 2 more hours.

Next, Fluorine-doped tin oxide (FTO) glass was cleaned by ultrasonication in acetone, ethanol, and deionized water for 20 min each and dried using nitrogen gas. The glass was placed into the mixed solution with the conductive side facing down, and the BiVO_4_ films were grown in a water bath at 65 °C, 75 °C, 85 °C, and 95 °C for 2 h. After the film’s growth, the FTO glass was transferred to a muffle furnace, where the temperature was ramped at 10 °C/min to 550 °C, with annealing occurring at this temperature for 2 h. The resulting films were labeled as 65-BiVO_4_, 75-BiVO_4_, 85-BiVO_4_, and 95-BiVO_4_ based on the growth temperature.

## 3. Results and Discussion

**Figure 1:**
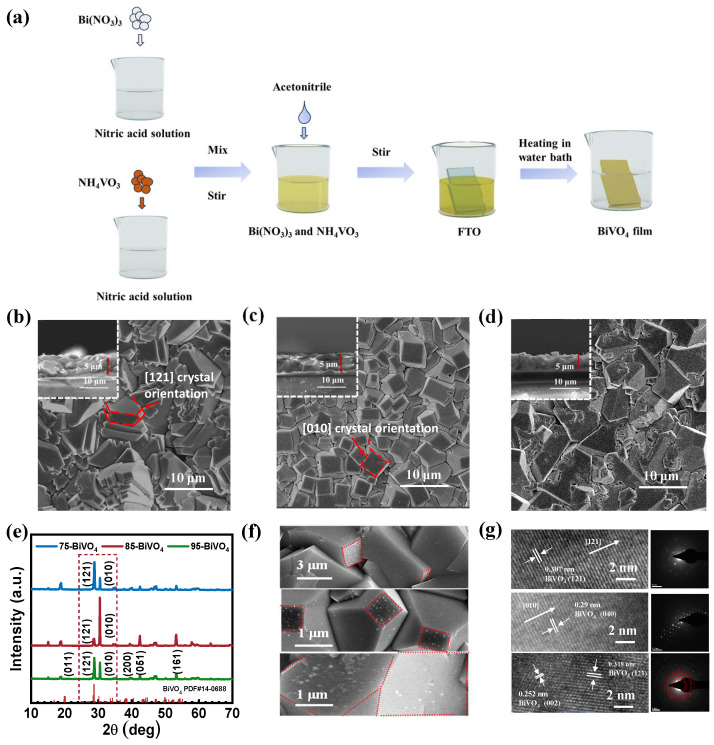
Synthesis and characterizations of BiVO_4_ catalytic films: (**a**) schematic illustration of the synthesis process; (**b**–**d**) top-view and cross-sectional SEM images of the BiVO_4_ films grown at water bath temperatures of 75 °C, 85 °C, and 95 °C, respectively; (**e**) XRD patterns; (**f**) SEM images of Pt photodeposited on BiVO_4_; (**g**) HR-TEM images and SAED pattern of typical sample.

The effect of water bath temperature on the crystal growth orientation of BiVO_4_ films was systematically investigated. As shown in Figure 1a, BiVO_4_ films with tunable crystal orientations were prepared by controlling the water bath temperature during the wet chemical process.

The corresponding top-view and cross-sectional images in Figure 1b–d demonstrate the growth of BiVO_4_ crystals on the FTO substrate at different temperatures. At 65 °C, BiVO_4_ crystals exhibit larger grain sizes and uneven growth (Figure S2). With increasing temperature, crystal density increases, resulting in dense, uniform film thickness of ∼5 μm. However, the crystal morphology and growth orientation differ across temperatures. At 75 °C, BiVO_4_ (75-BiVO_4_) forms decahedral structures with a 45° angle to the substrate, while at 85 °C, BiVO_4_ (85-BiVO_4_) grows parallel to the substrate with a more regular decahedral shape. In contrast, the 95 °C-grown BiVO_4_ (95-BiVO_4_) exhibits irregular morphology and no distinct growth orientation.

To further examine crystal structure and phase composition, X-ray diffraction (XRD) analysis was conducted. Figure 1e shows that all films correspond to monoclinic scheelite-type BiVO_4_ (JCPDS No. 14-0688), with varying diffraction peak intensities [25]. The 75-BiVO_4_ film exhibits the strongest (121) facet peak, while the 85-BiVO_4_ film shows the highest intensity for the (010) facet. The 95-BiVO_4_ film has similar intensities for both the (010) and (121) facets.

In the photodeposition experiment with chloroplatinic acid, due to the electron accumulation on the (010) facet of BiVO_4_, Pt particles preferentially deposit onto the (010) facet [26]. This experimental approach provides a clear basis for determining the exposed crystal facets and crystal growth orientation. As shown in Figure 1f, on the surface of the 75-BiVO_4_ film, Pt particles undergo oriented photodeposition along the crystal plane perpendicular to the FTO substrate. In contrast, in 85-BiVO_4_, Pt particles align parallel to the FTO substrate during photodeposition. This phenomenon distinctly reveals the orientation differences between the crystal planes of the 75-BiVO_4_ and 85-BiVO_4_ films. However, no oriented photodeposition of Pt particles was observed in the 95-BiVO_4_ film, indicating an absence of obvious facet exposure. Together with XRD analysis, these findings confirm that the 95-BiVO_4_ film lacks a preferred orientation in crystal growth.

As shown in Figure 1g, high-resolution transmission electron microscopy (HR-TEM) images of 75-BiVO_4_ and 85-BiVO_4_ reveal uniform lattice fringes, and the selected area electron diffraction (SAED) patterns further confirm that the growth directions of these grains are essentially consistent. However, 95-BiVO_4_ shows disordered lattice fringes and a ring-like SAED pattern, indicating that the grain growth directions are disordered. Moreover, the evident fringes observed in the high-resolution transmission electron microscopy (HR-TEM) images are basically perpendicular to the longitudinal direction of the nanoparticles. The lattice spacings of 75-BiVO_4_ and 85-BiVO_4_ are calculated to be 0.307 nm and 0.29 nm, respectively, which match well with the lattice spacings of the (121) and (040) planes of monoclinic BiVO_4_ with a space group of *I2/a*. Therefore, combined with XRD and photodeposition tests, it can be confirmed that the nanoparticles of 75-BiVO_4_ and 85-BiVO_4_ preferentially grow along the [121] and [010] directions, respectively.

Raman and XPS analyses (Figure S3) confirm the purity of the BiVO_4_ films, with no significant differences in defects or lattice distortions across different crystal orientations, ensuring that crystal properties aside from orientation remain consistent among the films [27,28].

**Figure 2:**
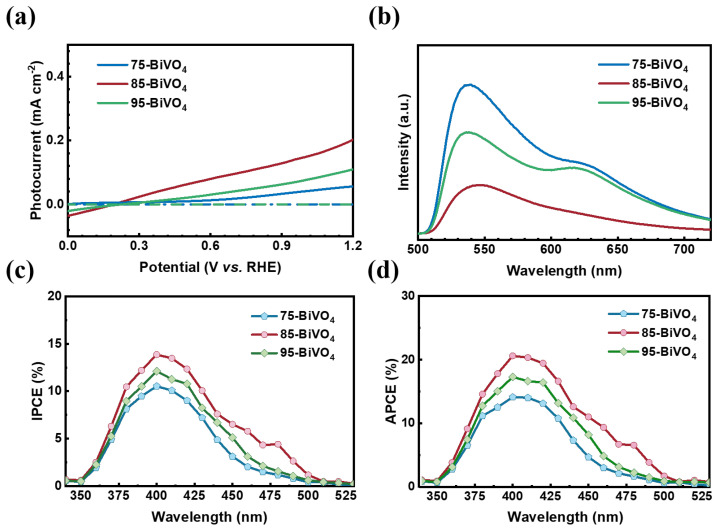
Photoelectrochemical characterization and comparison. (**a**) I-V curves. (**b**) PL spectra. (**c**) IPCE, and (**d**) APCE of three BiVO_4_ films with different crystal orientations.

To explore the intrinsic relationship between BiVO_4_ crystal orientation and PEC activity, a series of photoelectrochemical tests were conducted by using the BiVO_4_ films as photoanodes in a three-electrode system. First, linear sweep voltammetry (LSV) was performed in 0.5 M Na_2_SO_4_ electrolyte under AM 1.5 G Xe lamp illumination. As shown in Figure 2a, the [010]-oriented 85-BiVO_4_ photoanode achieved a photocurrent density of 0.2 mA cm^−2^, which is 3.5 times that of [121]-oriented 75-BiVO_4_ (0.056 mA cm^−2^) and double that of the randomly oriented 95-BiVO_4_ (0.11 mA cm^−2^). This highlights the significantly enhanced photocurrent density of the [010]-oriented BiVO_4_ photoanode.

Additionally, Figure 2b shows that under identical laser irradiation, the luminescence intensities of the BiVO_4_ films vary notably with crystal orientation. The [010]-oriented BiVO_4_ shows the lowest luminescence, while the [121]-oriented film exhibits the highest, indicating differences in electron–hole recombination rates [29]. Specifically, the [010]-oriented BiVO_4_ has the lowest radiative recombination rate, whereas the [121]-oriented film suffers from the highest recombination.

To further evaluate the wavelength dependence of PEC activity, the incident photon-to-current efficiency (IPCE) of the three films was measured under an applied bias of 1.2 V (vs. RHE) in 0.5 M Na_2_SO_4_ and 0.1 M Na_2_SO_3_ solutions [30]. Figure 2c shows that the [010]-oriented 85-BiVO_4_ exhibited higher IPCE values, with a rapid drop near 500 nm, consistent with its absorption edge. Even after calculating the absorbed photon-to-current efficiency (APCE) to account for absorption variations, the [010]-oriented BiVO_4_ still demonstrated superior PEC performance [31]. These findings indicate that BiVO_4_ films grown along the [010] orientation exhibit significantly better PEC activity compared to those grown along other orientations.

The photoelectrochemical activity of semiconductors is influenced by three key factors: light absorption rate, carrier separation, and injection efficiencies [32,33]. This study explores the differences in PEC performance of the three BiVO_4_ films based on these aspects.

**Figure 3:**
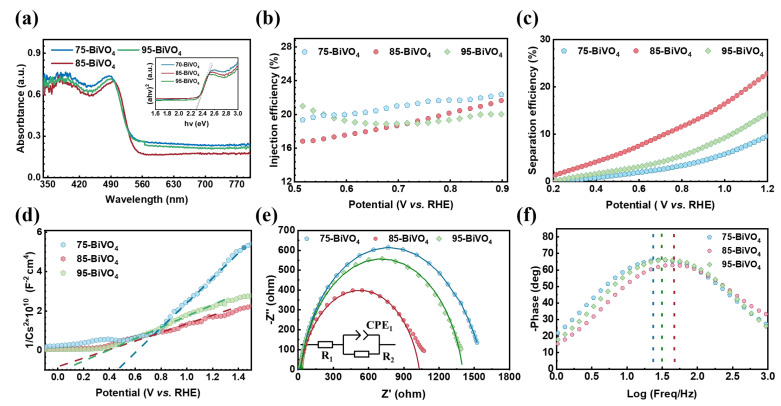
Band structure and photocarrier dynamics analysis. (**a**) UV-vis absorption spectra, with insets showing optical bandgap widths. (**b**) Injection efficiency. (**c**) Separation efficiency. (**d**) Mott–Schottky curves. (**e**) EIS. (**f**) Bode plots.

First, the optical properties of the BiVO_4_ films were analyzed using UV-vis spectroscopy. As shown in Figure 3a, the light absorption rates of the films with different crystal orientations are similar. All films have an absorption edge at 520 nm, with absorption rates of about 65% for wavelengths shorter than 520 nm and around 20% for longer wavelengths. The optical bandgap, calculated using the Tauc formula (as shown in Equation (1)) [34], is consistently 2.4 eV for all films, indicating minimal impact of crystal orientation on the optical properties.

(1) $${\left(\alpha h\nu \right)}^{1/n}=A\left(h\nu -E_{g}\right),$$

where α is the absorption coefficient and *n* is related to the type of transition of the semiconductor. If it is a direct bandgap semiconductor, $n=\frac{1}{2}$, and if it is an indirect bandgap semiconductor, n=2. By drawing the Tauc curve, the intersection point between the slope of the fitted curve and the X-axis is the optical bandgap of the semiconductor. The value of *n* is $\frac{1}{2}$ because they are all direct bandgap materials of BiVO_4_.

Next, the carrier transport characteristics of the BiVO_4_ films were investigated using photoelectrochemical testing. By adding 0.1 M Na_2_SO_3_ as a hole sacrificial agent to the 0.5 M Na_2_SO_4_ electrolyte, surface recombination of electron–hole pairs was eliminated. The [010]-oriented BiVO_4_ film exhibited a photocurrent density of 1.17 mA cm^−2^, significantly higher than the other crystal-oriented films. This suggests that crystal orientation affects carrier transport within the films.

To assess carrier separation, injection and separation efficiencies were calculated (as shown in Equations (2) and (3)). Figure 3b,c show that while injection efficiency was similar across the three films, the [010]-oriented BiVO_4_ exhibited a much higher separation efficiency. This indicates that the superior photocurrent density of the [010]-oriented BiVO_4_ is due to more effective carrier separation within the film.

(2) $$\eta_{\mathrm{sep}}=\frac{J_{{\mathrm{Na}}_{2}{\mathrm{SO}}_{3}}}{J_{0}},$$

(3) $$\eta_{\mathrm{inj}}=\frac{J}{J_{{\mathrm{Na}}_{2}{\mathrm{SO}}_{3}}},$$

where *J* is the actual photocurrent density (photocurrent density without sacrificial agent) in the 0.5 M Na_2_SO_4_ electrolyte, $J_{0}$ is the theoretical photocurrent, and $J_{{\mathrm{Na}}_{2}{\mathrm{SO}}_{3}}$ is the photocurrent density obtained in the 0.5 M Na_2_SO_4_ electrolyte with 0.1 M Na_2_SO_3_.

Further insights were gained from Mott–Schottky tests, which confirmed that all BiVO_4_ films are n-type semiconductors, regardless of their crystal orientation [35]. The carrier concentration of the [010]-oriented BiVO_4_ was 1.087 × 10^18^ cm^−3^, significantly higher than that of the 75-BiVO_4_ and 95-BiVO_4_ films. This confirms that fewer electron–hole pairs recombine in the [010]-oriented film, leading to higher carrier availability at the surface and superior photocurrent density.

Finally, electrochemical impedance spectroscopy (EIS) was used to examine carrier transport dynamics [36]. In this experiment, measurements were conducted under illumination. A constant voltage of 0.61 V (vs. RHE) was applied, with a frequency range of 0.1 Hz to 1000 kHz, an AC amplitude of 10 mV (vs. Ag/AgCl), and saturated Na_2_SO_4_ (0.5 M) solution as the electrolyte. The [010]-oriented BiVO_4_ film exhibited the lowest resistance and the shortest carrier lifetime (2.99 ms), demonstrating that carriers can be injected into the electrolyte more quickly, as shown in the Bode plot. These results demonstrate that this film has superior carrier transport properties, leading to enhanced separation efficiency, photocurrent density, and photoelectric conversion efficiency.

**Figure 4:**
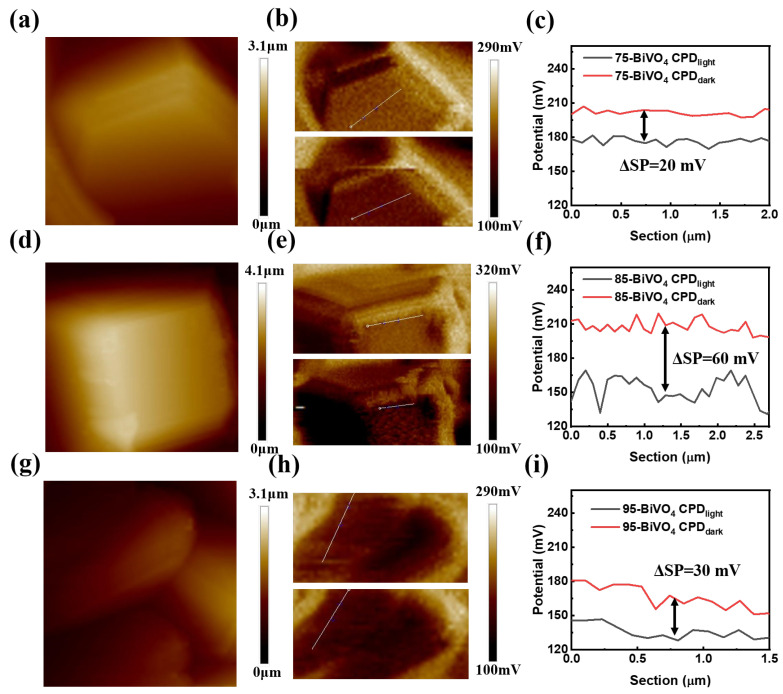
Surface potential measurements with KPFM. (**a**,**d**,**g**) Surface morphology images of 75-BiVO_4_, 85-BiVO_4_, and 95-BiVO_4_, respectively. (**b**,**e**,**h**) Surface potential mappings for the same samples, with the top row showing measurements in the dark and the bottom row showing measurements under UV illumination. (**c**,**f**,**i**) Surface potential distributions along the marked line in the corresponding surface potential images (**b**,**e**,**h**).

Kelvin probe microscope (KPFM) was employed to explore the surface morphology and surface potential distribution of individual BiVO_4_ crystal particles more intuitively [37,38,39]. As shown in Figure 4a,d,g, the morphological characteristics of monoclinic BiVO_4_ films with different crystal orientations are consistent with the SEM observations. These images clearly demonstrate the distinct surface features of the BiVO_4_ particles, which vary depending on the crystal orientation.

The influence of UV illumination on surface potential was also investigated, revealing important insights into carrier dynamics. As shown in Figure 4b,e,h, surface potential mappings under UV illumination (bottom images) exhibit a significant decrease compared to the dark conditions (top images). This reduction occurs because, when exposed to UV light, the Pt-coated probe with a higher work function causes photogenerated electrons to accumulate within the bulk of the BiVO_4_ particles, while photogenerated holes migrate to the surface. This shift results in a more positive surface potential for n-type semiconductors, reducing the measured contact potential difference (CPD) [40].

The extent of this surface potential change is closely related to carrier separation efficiency. As shown in Figure 4c,f,i, the surface potential difference (ΔSP) for 85-BiVO_4_ particles is 60 mV, significantly higher than the ΔSP values for 75-BiVO_4_ (20 mV) and 95-BiVO_4_ (30 mV). This suggests that the 85-BiVO_4_ crystal particles experience greater changes in surface Fermi level under illumination, indicating more effective separation of photogenerated carriers. As a result, more carriers reach the surface, leading to reduced carrier recombination within the 85-BiVO_4_ particles.

This enhanced carrier separation in 85-BiVO_4_ provides a clear explanation for its superior photoelectric performance, which is consistent with the earlier findings. These results further underscore the importance of crystal orientation in optimizing the photoelectrochemical properties of BiVO_4_ films.

**Figure 5:**
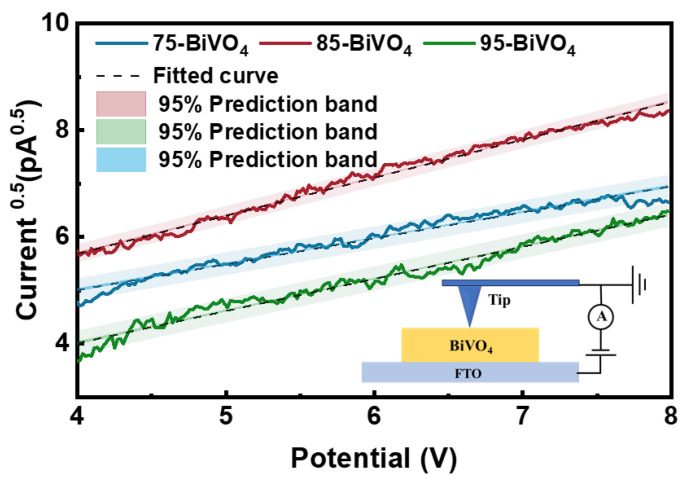
Local I–V curves measured with C-AFM.

To assess the influence of BiVO_4_ crystal growth orientation on internal carrier transport resistance, carrier mobility within BiVO_4_ crystal particles of varying orientations was measured using conductive atomic force microscopy (C-AFM). As shown in Figure 5, a high-work-function Pt-Ir coated probe was used as a movable nanoscale electrical contact point. When the probe contacts BiVO_4_ crystal particles, the difference in work functions results in a barrier. This allows for the application of the space-charge-limited current (SCLC) model to quantify carrier mobility in BiVO_4_. C-AFM offers high-resolution local I–V curve measurements with the added benefit of eliminating pinhole effects, providing valuable insights into carrier mobility. However, the geometry of the probe tip inevitably influences test results [41]. To account for this, the Johnson–Kendall–Roberts (JKR) contact model was employed to calculate the carrier mobility within different BiVO_4_ crystal particles [42,43]:

(4) $$J=\alpha \varepsilon \varepsilon_{0}\mu \frac{{\left(V-V_{bi}\right)}^{2}}{L^{3}}{\left(\frac{L}{d}\right)}^{1.6},$$

Here, *J* is the photocurrent density. α is a prefactor determined by Reid et al. through finite element modeling, with a value of 8.2, replacing the $\frac{9}{8}$ factor used in the Mott–Gurney law for planar electrodes. This adjustment eliminates errors caused by the non-uniform electric field at the probe tip. In this equation, ε is the relative dielectric constant of the film, $\varepsilon_{0}$ is the vacuum dielectric constant, μ is the carrier mobility, *V* is the applied voltage, and $V_{bi}$ is the built-in potential caused by the work function difference between the contact electrodes. *L* is the film thickness. To account for the probe tip shape, the factor ${\left(\frac{L}{d}\right)}^{1.6}$ is introduced, further reducing errors due to the curvature of the tip. Specific parameters can be found in Table S2.

Using Equation (4), the carrier mobility of the three BiVO_4_ photoanodes was calculated. The 85-BiVO_4_ shows a carrier mobility of 7.647 × 10^−3^ cm^2^ V^−1^ s^−1^, which is significantly higher than that of 75-BiVO_4_ (3.572×10^−3^ cm^2^ V^−1^ s^−1^) and 95-BiVO_4_ (4.988×10^−3^ cm^2^ V^−1^ s^−1^). This increased carrier mobility in 85-BiVO_4_ indicates more efficient photogenerated charge transport, contributing to its superior PEC performance, especially along the [010] crystal orientation.

## 4. Conclusions

In conclusion, this study successfully fabricated BiVO_4_ films with distinct crystal orientations via a wet chemical method and systematically investigated their photoelectrochemical properties. The films grown along the [010] crystal orientation demonstrated notably superior PEC performance, which can be attributed to reduced carrier recombination and enhanced charge separation efficiency. Surface potential measurements and conductive atomic force microscopy revealed that the [010]-oriented BiVO_4_ films possess higher carrier mobility, leading to lower internal transport resistance and more efficient charge transport to the surface. This enhanced mobility was confirmed to play a pivotal role in the observed improvements in PEC activity. Furthermore, this study highlights the critical role of crystal facet engineering in optimizing semiconductor performance for PEC applications. By orienting crystal growth along specific facets, such as [010], it is possible to increase carrier mobility and improve overall photocatalytic efficiency. These findings provide valuable insights for developing advanced photoelectrodes with higher carrier transport efficiency, paving the way for more effective solar energy conversion and environmental remediation technologies. Further research could explore the effects of other crystal orientations and multi-faceted BiVO_4_ structures, potentially leading to even more efficient photoanodes for solar energy conversion. Additionally, combining this strategy with other co-catalysts or hybrid materials could significantly boost PEC performance for practical applications. 

## Data Availability

The original contributions presented in the study are included in this article; further inquiries can be directed to the corresponding author.

## Supplementary Materials

The following supporting information can be downloaded at: https://www.mdpi.com/article/10.3390/nano14231870/s1, the instruments and their corresponding models used in this paper, the specific testing parameters, the formulas used for calculations, and some supplementary data. Figure S1: Top-view SEM images of BiVO_4_ films grown for different durations in a water bath at 85 °C. Figure S2: SEM top-view images (a) and (b), as well as a cross-sectional view (c) of BiVO_4_ films grown in a water bath at 65 °C. Figure S3: Raman spectra (a) and XPS survey spectra (b) of the three films. Figure S4: (a) The I-t analysis of the BiVO_4_ films under light on/off cycles and (b) the stability test of the BiVO_4_ films under continuous illumination. Figure S5: XRD images of (a) 75-BiVO_4_, (b) 85-BiVO_4_, and (c) 95-BiVO_4_ photoanodes before and after stability testing. Figure S6: SEM images of (a) 75-BiVO_4_, (c) 85-BiVO_4_, and (e) 95-BiVO_4_ photoanodes before stability testing, along with SEM images of (b) 75-BiVO_4_, (d) 85-BiVO_4_, and (f) 95-BiVO_4_ photoanodes after stability testing. Figure S7: Electrochemical active surface area (ECSA) measurements, including cyclic voltammetry (C-V) curves of (a) 75-BiVO_4_, (b) 85-BiVO_4_, and (c) 95-BiVO_4_ photoanodes, as well as (d) the plots of capacitive current to scan rate. Figure S8: I–V characteristics measured in a mixed electrolyte of 0.5 M Na_2_SO_4_ and 0.1 M Na_2_SO_3_ under (a) illuminated and (b) dark conditions. Figure S9: Local I–V curves measured by conductive atomic force microscopy (C-AFM). Table S1: Summary of the slope and carrier concentrations. Table S2: Carrier mobility determined from the local I-V curves. Table S3: The fitting results of EIS Nyquist spectra.
