# Supplementary material for: Enhancing Photoelectrocatalytic Efficiency of BiVO4 Photoanodes by Crystal Orientation Control
Source: Nanomaterials (Basel). 2024 Nov 21;14(23):1870. doi: 10.3390/nano14231870 (PMC11643834; doi:10.3390/nano14231870)
Supplement: Supplementary file 1 [file nanomaterials-14-01870-s001.zip › nanomaterials-3308312-supplementary.pdf]

Enhancing photoelectrocatalytic efficiency of $BiVO_4$ photoanodes by crystal orientation control

Hongru Zhao,[1,2] Xinkong Wei,[2] Yue Pei,[2] Weihua Han[1,2]*

1 Guangzhou Institute of Blue Energy, Guangzhou 510555, China

2 School of Physical Science and Technology, Lanzhou University, Lanzhou 730000, China

*Corresponding Author

E-mail: hanwh@lzu.edu.cn (Weihua Han)

Characterization

The morphology evolution in various cases was investigated using scanning electron microscopy (SEM, Apro S) and transmission electron microscopy (TEM, FEI Tecnai F30). X-ray photoelectron spectroscopy (XPS, Kratos AXIS Ultra DLD XPS instrument, Al Ka source, hv = 1486.6 eV) is used to analyze the changes in chemical element composition and chemical state of the sample. The crystal structure and surface composition were performed by X-ray diffractometer (XRD, Philips, X' per pro, Cu Kα 0.154056 nm) and micro-Raman spectrometer (Jobin-Yvon LabRam HR800 spectroscope, 532 nm laser). A 550 W Xenon lamp with an AM 1.5G filter was used to simulate solar illumination. The incident light intensity was calibrated to 100 $mW\cdot cm^{-2}$ by a standard silicon solar cell. The sample was used as the working electrode, the platinum sheet and Ag/AgCl (saturated KCl) as the counter electrode and the reference electrode, and the electrolyte used during the test was a saturated Na_2SO_4 solution (0.5 M). Where the scanning rate of linear sweep voltammetry (LSV) is 20 mV/s, the relationship between sample current density and voltage (*I-V*) can be obtained. Electrochemical impedance spectroscopy (EIS) and Bode plots can be used to indirectly indicate the magnitude of impedance and carrier lifetime during carrier transport, where the constant voltage applied is 0.3 V (*vs.* Ag/AgCl), the frequency range is 0.1 Hz to 1000 KHz, the amplitude is 10 mV (*vs.* Ag/AgCl), and the electrolyte is saturated Na_2SO_4 (0.5 M) solution. This study utilized Atomic Force Microscopy (AFM, Bruker Dimension Icon) to characterize the morphology and photoelectric properties of the photoelectrodes. The AFM, KPFM, and C-AFM tests were conducted using SCM-PIT-V2 probes.

Equations used in this work

1. Charge separation efficiency and surface charge injection efficiency

The carrier dynamics determine the PEC properties of photocatalyzed films. The carrier separation efficiency (η_{sep}) and the interface injection efficiency (η_{inj}) of the carrier can be obtained by LSV test. The η_{sep} and η_{inj} of the sample can be calculated using the following equations (S1) - (S5):

$$J = J_0 \cdot \eta_{sep} \cdot \eta_{inj} \ , \tag{S1}$$

where J is the actual photocurrent density (Photocurrent density without sacrificial agent) in 0.5 M Na_2SO_4 electrolyte, J_0 is the theory photocurrent calculated by measuring the light absorption of catalyst and integrating it with respect to the AM 1.5 G solar spectrum:

$$J_0 = \int qA\phi \, d\lambda \, , \tag{S2}$$

where q is the elementary charge of electron, A (%) is the light absorption of the sample at the corresponding wavelength λ, and ϕ is the photon flux of the AM 1.5G standard spectrum, we used Na_2SO_3 as the electrolyte solution, which can also act as a hole captor. It can promote the rapid transfer of photogenerated holes to the electrolyte, reduce the recombination of photogenerated holes and photogenerated electrons, and increase the lifetime of photogenerated electrons, when there are sacrificial agents (Na_2SO_3) in the electrolyte, the interface injection efficiency is approximately 100%.

$$J_{Na_2SO_3} = J_0 \cdot \eta_{sep} \cdot \eta_{inj} \ , \tag{S3}$$

$$\eta_{sep} = \frac{J_{Na_2SO_3}}{J_0} \, , \tag{S4}$$

$$\eta_{inj} = \frac{J}{J_{Na_2SO_3}} \, , \tag{S5}$$

$J_{Na_2SO_3}$ are the photocurrent densities obtained in 0.5 M Na_2SO_4 electrolyte with 0.1 M Na_2SO_3.

2. Kubelka − Munk function

The Kubelka − Munk function can be described as the following equation (S6):

$$(\alpha h\upsilon)^{\frac{1}{n}} = A\left(h\upsilon - E_g\right) \, , \tag{S6}$$

where α is the absorption coefficient and n is related to the type of transition of the semiconductor, if it is a direct bandgap semiconductor, $n = 1/2$, if it is an indirect bandgap semiconductor, $n = 2$. By drawing the Tauc curve, the intersection point

between the slope of the fitted curve and the X-axis is the optical band gap of the semiconductor. The value of n is $1/2$, because they are all direct band gap materials of $BiVO_4$.

3. The lifetime of photogenerated charge (τ_e)

The lifetime of the photogenerated charge was calculated using the equation (S7):

$$\tau_e = 1/2\pi f_{max} , \tag{S7}$$

where f_{max} is at the middle frequency peak appears in the Bode-phase plot.

4. Mott-Schottky Test

The Mott-Schottky curve (M-S curve) can reflect the P/N characteristics of the sample, and can also calculate the carrier concentration (N_d) and the band potential (E_{fb}) in the sample, as shown in equation (S8). If the slope is $K > 0$ indicates that the sample is an N-type semiconductor, conversely, it is a P-type semiconductor. The smaller the absolute value of the measured curve slope, the greater the carrier concentration. The point at which the tangential extension in the inverse region of the M-S curve intersects the X-axis is the flat-band potential (E_{fb}) of the sample.

$$\frac{1}{C_{sC}^2} = \frac{2}{\varepsilon_0 \varepsilon_r q N_d}\left(E - E_{fb}\right) , \tag{S8}$$

Where N_d is the carrier concentration, q is the charge of the electron (1.6×10^{-19} C), ε_0 is the vacuum dielectric constant (8.85×10^{-12} Fm^{-1}), ε_r is the relative dielectric constant of the sample, the AC frequency is chosen to be 2500 Hz, Mott-Schottky's hypothesis will be satisfied in this frequency range. Too high a frequency exacerbates the effect of noise, while too low a frequency can mask other series capacitors.

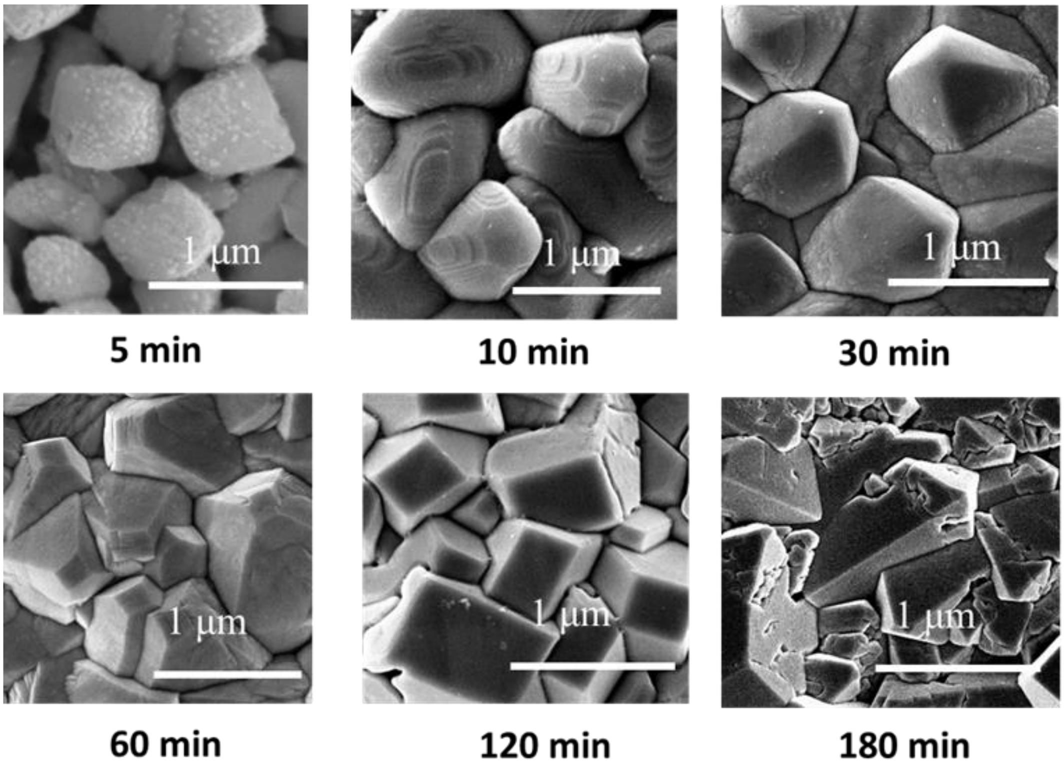

Fig. S1: Top-view SEM images of $BiVO_4$ films grown for different durations in a water bath at 85°C.

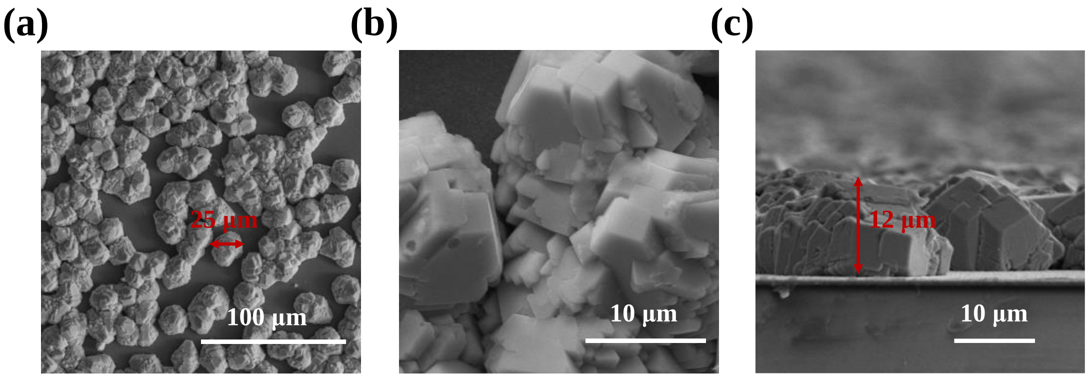

Fig. S2: SEM top-view images (a) and (b), and a cross-sectional view (c) of $BiVO_4$ films grown in a water bath at 65°C.

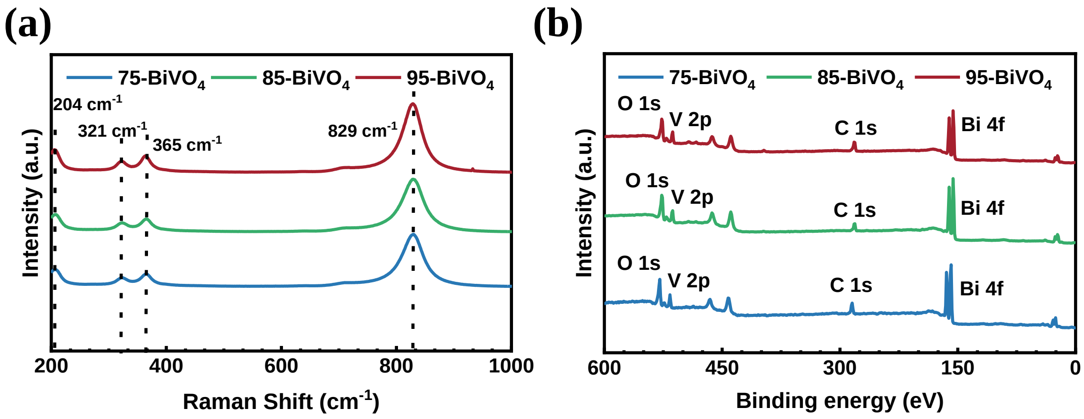

Fig. S3: Raman spectra (a) and XPS survey spectra (b) of the three films.

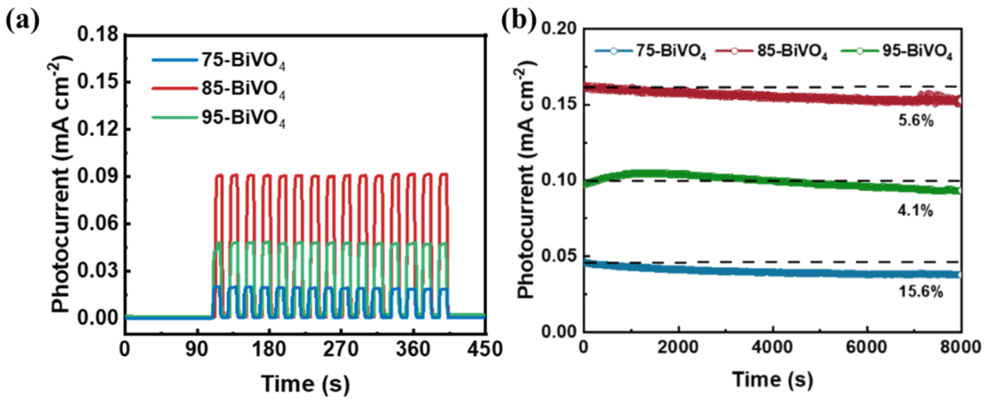

Fig. S4: (a) *I-t* analysis of the $BiVO_4$ films under light on/off cycles. (b) Stability test of the $BiVO_4$ films under continuous illumination.

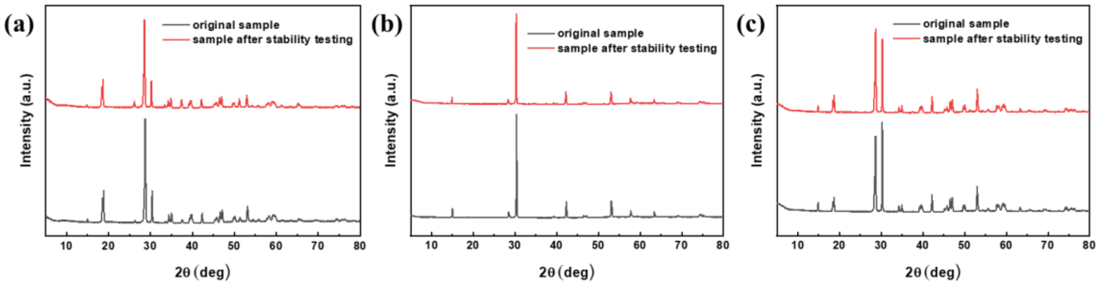

Fig S5 The XRD images of (a) 75-$BiVO_4$, (b) 85-$BiVO_4$, and (c) 95-$BiVO_4$ photoanodes before and after stability testing.

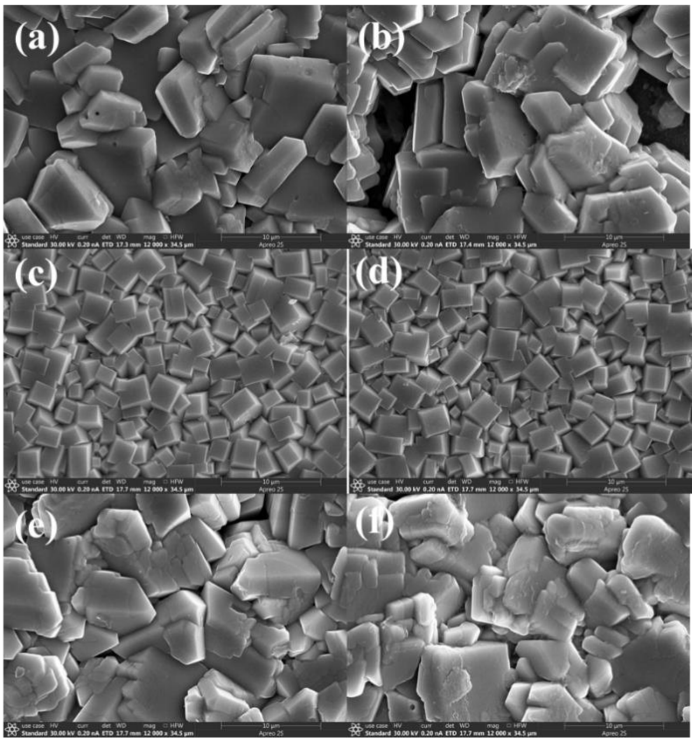

Fig S6 The SEM images of (a) 75- $BiVO_4$, (c) 85- $BiVO_4$, and (e) 95- $BiVO_4$ photoanodes before stability testing, and the SEM images of (b) 75- $BiVO_4$, (d) 85- $BiVO_4$, and (f) 95- $BiVO_4$ photoanodes after stability testing.

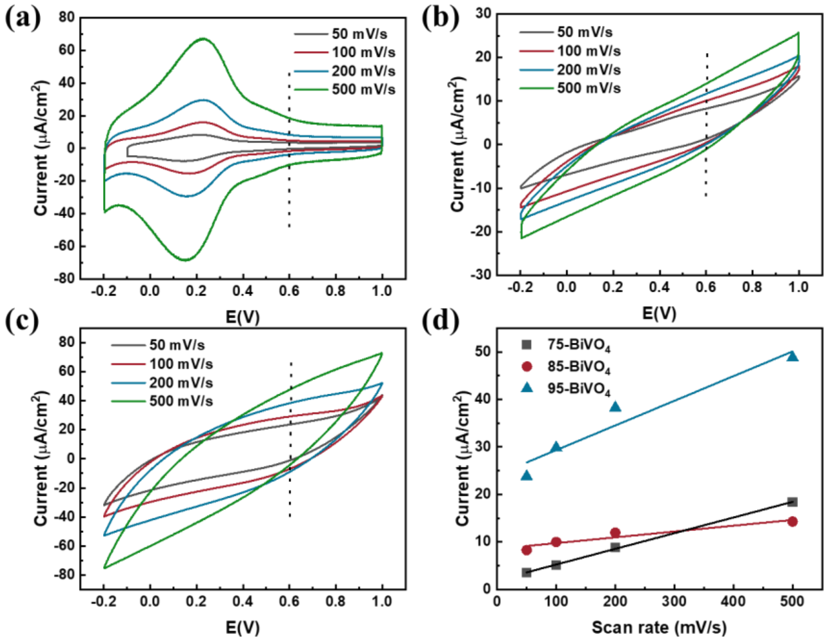

Fig S7 Electrochemical active surface area (ECSA)measurements. Cyclic voltammetry (C-V) curves of (a) 75-$BiVO_4$, (b) 85-$BiVO_4$ and (c) 95-$BiVO_4$ photoanodes. (d) The plots of capacitive current to scan rate.

By calculating the slopes of the capacitive current versus scan rate for the 75-$BiVO_4$, 85-$BiVO_4$, and 95-$BiVO_4$ photoanodes, the relative electrochemical active surface areas (ECSA) of these photoanodes were determined to be 3.3:1.2:5.2. Among them, the 85-$BiVO_4$ photoanode has the smallest relative ECSA, yet it still demonstrates a relatively high photocurrent density.

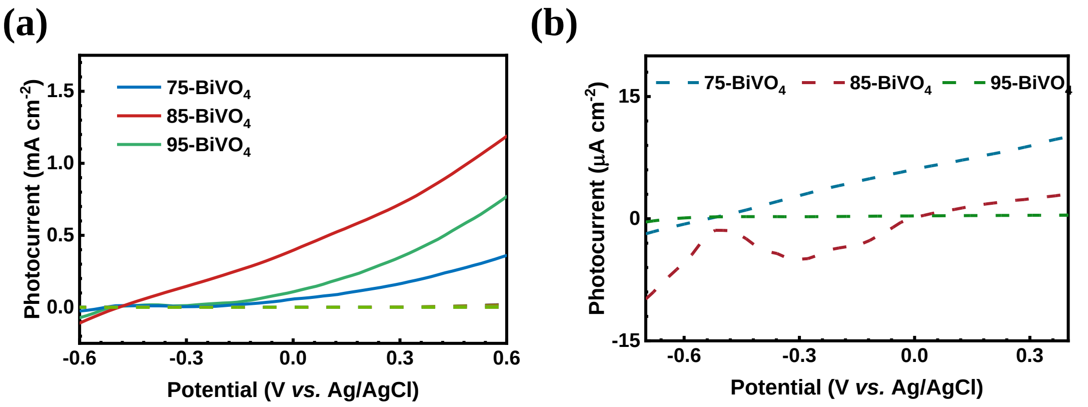

Fig. S8: *I-V* characteristics measured in a mixed electrolyte of 0.5 M Na_2SO_4 and 0.1 M Na_2SO_3 under illuminated (a) and dark (b) conditions.

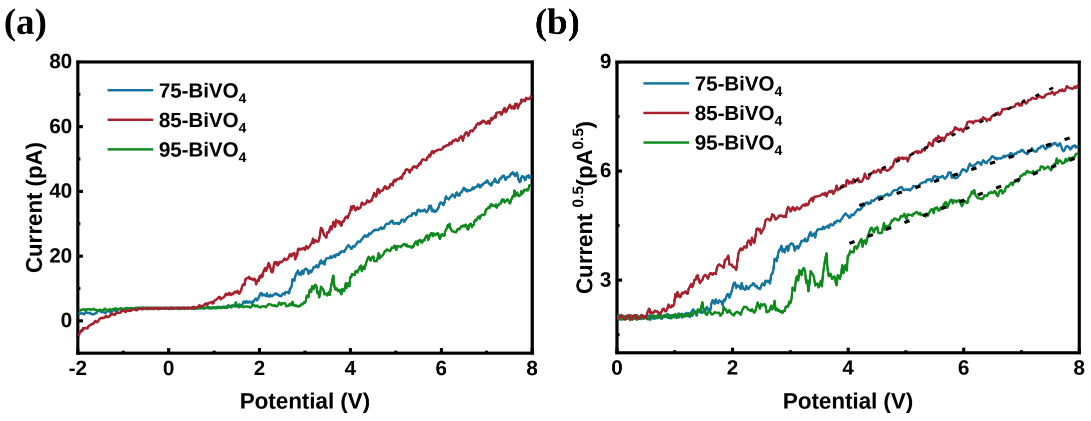

Fig. S9: Local *I-V* curves measured by C-AFM.

Table S1. The slope and carrier concentrations.

Photocathode	slope	N_d (cm^{-3})
**75-$BiVO_4$**	6.44×10^{10}	3.224×10^{17}
**85-$BiVO_4$**	1.91×10^{10}	1.087×10^{18}
**95-$BiVO_4$**	2.77×10^{10}	7.497×10^{17}

Table S2. The carrier mobility was determined from the local *I-V* curves.

Photocathode	L (m)	d (m)	V_b (V)	S (m^2)
**75-$BiVO_4$**	5×10^{-6}	2.5×10^{-9}	0.18	1.56×10^{-18}
**85-$BiVO_4$**	5×10^{-6}	2.5×10^{-9}	0.15	1.56×10^{-18}
**95-$BiVO_4$**	5×10^{-6}	2.5×10^{-9}	0.13	1.56×10^{-18}

Table S3. The fitting results of EIS Nyquist spectra.

Photocathode	75-$BiVO_4$	85-$BiVO_4$	95-$BiVO_4$
**R_1 (Ω)**	13.03	23.2	17.589
**R_2 (Ω)**	1604.1	1010.5	1383.6
